# Bax Targeted by miR-29a Regulates Chondrocyte Apoptosis in Osteoarthritis

**DOI:** 10.1155/2019/1434538

**Published:** 2019-03-12

**Authors:** Guiqiang Miao, Xuehui Zang, Huige Hou, Hui Sun, Lihui Wang, Ting Zhang, Yongtao Tan, Wenzhou Liu, Pei Ye, Lihua Gao, Zhengang Zha

**Affiliations:** ^1^Department of Orthopedics, The First Affiliated Hospital of Jinan University, Guangzhou 510630, China; ^2^Department of Orthopedics, Nanhai Hospital of Southern Medical University and Foshan Nanhai District People's Hospital, Guangdong 528200, China

## Abstract

Osteoarthritis (OA) is a chronic degenerative joint disease, where chondrocyte apoptosis is responsible for cartilage degeneration. Bax is a well-known proapoptotic protein of the Bcl-2 family, involved in a large number of physiological and pathological processes. However, the regulation mechanisms of Bax underlying chondrocyte apoptosis in OA remain unknown. In the present study, we determined the role of Bax in human OA and chondrocyte apoptosis. The results showed that Bax was upregulated in chondrocytes from the articular cartilage of OA patients and in cultured chondrocyte-like ATDC5 cells treated by IL-1*β*. Bax was identified to be the direct target of miR-29a by luciferase reporter assay and by western blotting. Inhibition of miR-29a by the mimics protested and overexpression by miR-29a inhibitors aggravated ATDC5 apoptosis induced by IL-1*β*. These data reveal that miR-29a/Bax axis plays an important role in regulating chondrocyte apoptosis and suggest that targeting the proapoptotic protein Bax and increasing expression levels of miR-29a emerge as potential approach for protection against the development of OA.

## 1. Introduction

Osteoarthritis (OA) is a common degenerative disease of the human articular cartilage and subchondral bone, characterized by destruction of articular cartilage, formation of osteophytes, synovitis, and intra-articular inflammation [1, 2]. Its clinical symptoms include pain, stiffness, and loss of mobility. Variable factors have been indicated to be responsible for the risk of OA, such as aging, failure of nutrient supply, and joint injury, and chondrocyte loss has been implicated as one key event in the development of OA [3]. Compared to normal cartilage, patients with OA show chondrocyte carrying apoptotic features [4, 5]. It is known that interleukin-1beta (IL-1*β*) plays pivotal roles in the pathogenesis of OA [6]. IL-1*β* activation induces apoptosis of chondrocytes in both normal and OA cartilage in a dose-dependent manner [5]. IL-1*β* treatment causes mitochondrial dysfunction and energy depletion in chondrocyte-like ATDC5 cell [7]. Moreover, IL-1*β* treatment increases the expression level of proapoptotic Bcl-2 family proteins [8, 9]. Here, IL-1*β* induced cell model was selected to reveal the mechanisms of chondrocyte apoptosis in the current study.

Bax, a proapoptotic protein of the Bcl-2 family, is critical for execution of apoptosis [10]. Upregulation and activation of Bax have been determined in many apoptotic models, such as androgen stimulated osteoblast and osteocyte apoptosis [11], BMP-2 induced osteoblast apoptosis [12], recombinant IL-1*β* induced cell cycle arrest, and apoptosis in neural precursor cells [13] and in cancer cell apoptosis [14, 15]. Genetic knockdown or knockout of Bax significantly suppresses cellular apoptosis, indicating the importance of the conserved mechanism of Bax-mediated cell apoptosis. However, the regulation mechanisms of Bax underlying chondrocyte apoptosis in OA remain to be explored.

MicroRNAs (miRNAs or miRs) are a family of ~22-nucleotide small, single stranded noncoding RNAs that regulate multiple genes by mediating mRNA cleavage and destabilization, involved in a variety of physiological functions and disease processes, including OA [16, 17]. It is known that miRNAs play an important role in mediating the effect of many risk factors for OA, such as aging [18], immune response [19, 20], pain [21], and inflammation [22, 23]. Collective data indicate that miRNA may play a critical role in regulating chondrocyte apoptosis in OA cartilage. miR-146a contribute to OA pathogenesis by inducing chondrocyte apoptosis via Smad4 [24]. Silencing of miR-34a reduces IL-1*β* induced chondrocyte apoptosis in vitro [25]. Besides, many other miRNAs were involved in the pathogenesis of OA, such as miR-448 [26], miR-21 [27], and miR-142-3p [28]. Moreover, the level of miR-29a in knee cartilaginous tissue of OA patients was significantly decreased, compared with normal cartilage samples [29], indicating the importance of miR-29a in the development of OA.

The role and detailed regulation mechanisms of Bax in OA remain to be elucidated. Whether miRNA could regulate Bax expression that contributes to chondrocyte apoptosis is largely unknown. Therefore, this work aims to elucidate the role of Bax, regulated by miRNA, in regulating chondrocyte apoptosis and pathogenesis of OA.

## 2. Material and Methods

### 2.1. Human Articular Chondrocyte Culture

Human articular cartilage samples were obtained from 7 patients with femoral head or tibia plateau fracture (age range: 68-82 years old) with clear history of joint pain, or from 11 patient (age range: 61-76 years old) with OA. Informed written consent was signed and all the procedures were approved by the Ethics Committee Board of the First Affiliated Hospital of Jinan University. For cell culture, cartilage samples were chopped into pieces and digested with collagenase D. Then cells were maintained at 37°C with 5% CO_2_ in Dulbecco's Modified Eagle Medium (DMEM, Carlsbad, CA, USA) containing 10%* v/v* fetal bovine serum (FBS, Invitrogen) and 1%* v/v* of penicillin/streptomycin.

### 2.2. Immunohistochemistry

Immunohistochemistry for Bax was performed. Briefly, fresh human cartilage samples were dissected and fixed in 4% paraformaldehyde overnight at 4°C. Then samples were embedded in paraffin and cut into sections with 5 *μ*m. Sections then were deparaffinized in xylenes and rehydrated in graded alcohols. Endogenous peroxidase was inactivated by 3% H_2_O_2_ in methanol for 10 min. After antigen retrieval, sections were incubated with an anti-Bax antibody (1:300, Abcam, Cambridge, MA, USA) at 4°C overnight. After washing with PBS, the expression of Bax was revealed by a polymer reagent by diaminobenzidine tetrahydrochloride (DAB). The expression level of Bax was captured using BX51 microscope (Olympus, Tokyo, Japan) and analyzed by IPP software (Media Cybernetics, Silver Spring, MD, USA).

### 2.3. ATDC5 Cell Cultures

The murine chondrogenic ATDC5 cell line (ATCC, Manassas, VA, USA) was obtained in DMEM/F-12 (Invitrogen), supplemented with Glutamine (2 mM, Invitrogen) and 10% FBS, and then incubated at 37°C with 5% CO_2_. For differentiation induction, cultured medium was supplemented with ITS (containing Insulin, Transferrin and Selenous Acid; from Cyagen Bioscience, Guangzhou, China) for 2 weeks. Differentiation of ATDC5 cells into chondrocyte-like cells was confirmed as previously reported [7, 30].

### 2.4. Apoptotic Assay

Chondrocyte-like ATDC5 cells were stressed with 10 ng/ml IL-1*β* (Sigma, St. Louis, MO, USA) for 48 h and stained with a permeable dye, Hoechst 33258 (Invitrogen, 5 *μ*g/ml). Then nuclear morphology was revealed, and images were captured by the fluorescence BX51 microscope. Cells with condensed nuclei were identified as apoptotic cells. The percentage of apoptotic cells was calculated from at least of 8 fields in an unbiased manner.

### 2.5. Western Blotting

Western blotting was applied as previous reported [31]. Briefly, cell lysates were collected, and protein concentration was determined by Bradford assay. Then 20 *μ*g of total protein from each sample was separated by SDS-polyacrylamide gel electrophoresis (PAGE). Then proteins were transferred onto PVDF membranes, blocked with 5% BSA for 1 hour, and incubated overnight at 4°C with primary antibodies against Tubulin, Bax, PUMA, total caspase-3, or cleaved (activated) caspase-3 (all purchased from Cell Signaling Technology, Danvers, MA, USA). After washing, membranes were incubated with secondary antibodies (Jackson ImmunoResearch, West Grove, PA, USA) for 60 min and bands were visualized using ECL chemiluminescence system (Beyontime, Zhenjiang, Jiangsu, China). Densitometric analysis was performed using ImageJ software (1.47v, US National Institutes of Health, USA). Band intensities were normalized to Tubulin in control group.

### 2.6. miRNA, 3'UTRs, and Luciferase Reporter Assay

DNA fragments containing miR-29a hairpin were cloned into IRES-GFP vector (Promega, Madison, WI, USA) for overexpression. Wildtype (miR-29a-WT) and mutated (miR-29a-MUT) miR-29a were shown in Figure 3(f). The sequence of Bax 3'UTR within miR-29a binding site was subcloned into pMiR-luciferase report vector (Promega). Dual-Luciferase® Reporter Assay System (Promega, Madison, WI, USA) was employed to measure the luciferase activities after transfection, accordingly [32]. Wildtype (Bax-WT) and mutated (Bax-MUT) bax were shown in Figure 3(f). miRNA transfection control, negative control, miR-29a mimic, and inhibitor were purchased from Genepharma (Shanghai, China).

### 2.7. Quantitative Real-Time PCR (qPCR)

Total RNA was isolated by TRIzol (Invitrogen). For miRNA extraction, reverse transcription was performed with TaqMan MicroRNA Reverse Transcription Kit (Applied Biosystems, Carlsbad, CA, USA), according to the instruction. Then PCR reactions were conducted with predesigned primers for miR-29. The following sense and antisense primers were used: miR-29a 5′-CTGATTTCTTTTGGTGTTCAG-3′ 5′-AACCGATTTCAGATGGTGC-3′; miR-29b 5′-CATATGGTGGTTTAGATTT-3′ 5′-AACACTGATTTCAAATGGT-3′; miR-29c 5′-CGATTTCTCCTGGTGTTCA-3′ 5′-ACCGATTTCAAATGGTGC-3′; *β*-actin 5′-AAATCTGGCACCACACCTTC-3′ 5′-GGGGTGTTGAAGGTCTCAAA-3′.

### 2.8. Statistical Analysis

Data represent as means ± SD from at least 3 independent experiments for n > 3 cultures. Statistical analyses were performed by Graphpad Prism 7.0 software (GraphPad Software Inc., La Jolla, USA). The P-values were calculated by using a two-way analysis of variance (ANOVA) followed by Bonferroni test (for more than two groups), or two-tailed student's* t*-test (within two groups). A value of* p* < 0.05 was considered significant.

## 3. Results

### 3.1. Bax Is Upregulated in the Cartilages of OA Patients and in Cultured Chondrocytes from OA Patients

**Figure 1:**
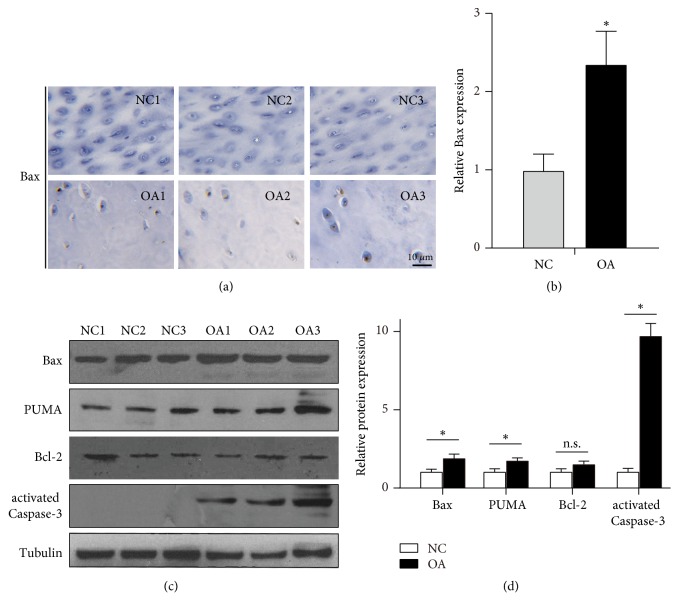
*Bax is upregulated in chondrocytes from articular cartilage samples of OA patients*. (a) Histological sections of articular cartilage samples of OA or control (NC) patients were immunostained using anti-Bax antibody. Representative images were shown, and the statistical analyses were calculated and shown in (b). (c) Cultured human chondrocytes from clinical samples were subjected to western blotting with anti-Bax, anti-PUMA, anti-Bcl-2, and anti-activated Caspase-3 antibodies. Tubulin was used as loading control. Relative expressions were evaluated, and the statistical data were shown in (d). *∗* donates* p* < 0.05 and n.s. donates no significant difference, compared to NC group.

Although known as the toxic proapoptotic protein, the role of Bax in chondrocyte apoptosis is less known, especially the detailed mechanisms. Thus, we first determined the expression level in the cartilage isolated from OA patients, by immunohistochemical staining. When compared to the normal control, the expression of Bax was significantly upregulated in the chondrocyte of OA patients (Figure 1(a)). The staining was quantified, and data showed that Bax level was found increased more than 2-fold in OA patients (Figure 1(b)). Further, chondrocytes from clinical samples were isolated and cultured. By comparing to control chondrocytes, cells from OA patients showed remarkedly upregulated Bax protein level (Figure 1(c)). PUMA, another proapoptotic protein as Bax, was reported to be involved in the regulation of OA [8]. Accordingly, the expression level of PUMA was also upregulated revealed by western blot analysis (Figure 1(c)). On the other side, the levels of antiapoptotic protein Bcl-2 were also evaluated and showed no statistical differences (Figure 1(c)). Furthermore, the levels of activated Caspase-3 were also upregulated in cells from OA samples (Figure 1(c)), as reported showing apoptotic features [4, 5]. The statistical data were shown in Figure 1(d). Taken together, these data showed that Bax protein level was significantly upregulated in the chondrocytes in OA patients.

### 3.2. Bax Is Upregulated in Chondrocyte-Like ATDC5 Cells under IL-1*β* Treatment

**Figure 2:**
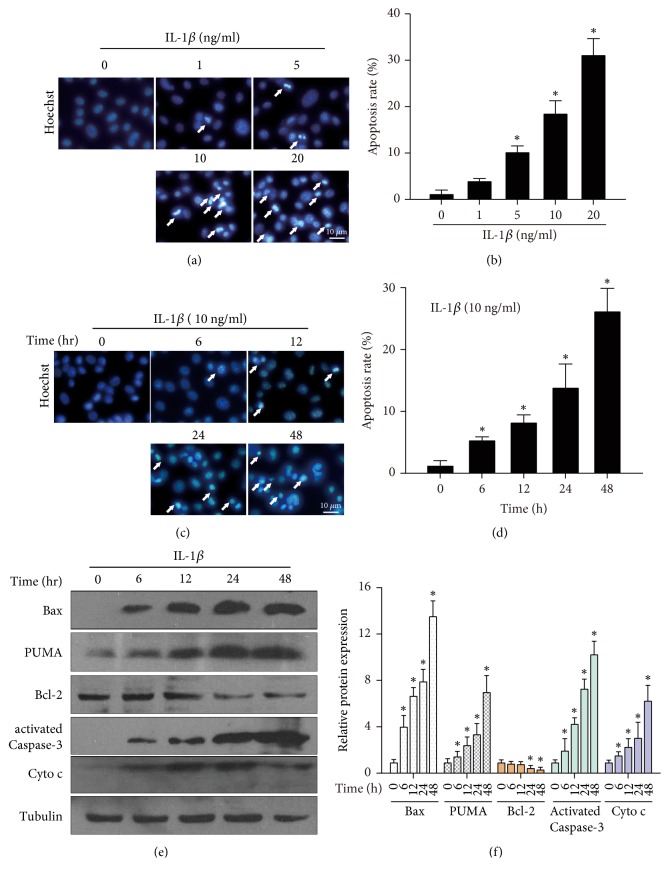
*Upregulation of Bax in IL-1β induced chondrocyte-like ATDC5 cell apoptosis*. ATDC5 cell was cultured and supplemented with ITS for two weeks. Then cells were treated with 1, 5, 10, and 20 ng/ml IL-1*β* for 24 h. Cells were stained with Hoechst 33258 for 10 min and the representative images were shown in (a). The apoptotic rate was counted and shown in (b) and *∗* donates* p* < 0.05, compared to 0 group. Cells treated with 10 ng/ml IL-1*β* for 6, 12, 24, and 48 h and representative images were shown in (c) and statistical data were shown in (d) and *∗* donates* p* < 0.05, compared to 0 group. (e) Lysate of cell treated in (c) was subjected to western blot with Bax, PUMA, Bcl-2, Cytochrome c (Cyto c), and active caspase-3 antibodies. Tubulin was stained with loading control. The relative expression levels were shown in (f). *∗*, #, and & donate* p* < 0.05, compared to 0 group.

To further explore the regulation of Bax in OA, we cultured ATDC5 as described in the material and method section. Then the chondrocyte-like ATDC5 cells were incubated with IL-1*β* to mimic the pathogenesis of human OA chondrocytes [5]. We first determined whether IL-1*β* exposure would induce ATDC5 apoptosis, by using nuclei staining. As shown in Figure 2(a), treatment of IL-1*β* for 24 h significantly induced ATDC5 apoptosis with increased condensed or fragmented nuclei, in a dose dependent manner (Figure 2(b)). Also, IL-1*β* exposure induces ATDC5 apoptosis in a time dependent manner (Figures 2(c) and 2(d)). Next, the expression levels of Bax were determined. As shown in Figure 2(e), treatment of IL-1*β* significantly induced the expression of Bax, in a time dependent manner. Meanwhile, as previously shown, PUMA was also increased after IL-1*β* treatment. The expression levels of Bcl-2 were downregulated upon IL-1*β* treatment and the expression levels of apoptotic activated Caspase-3 and Cytochrome c were markedly increased (Figures 2(e) and 2(f)). These data indicate that IL-1*β* exposure induces chondrocyte-like ATDC5 cell apoptosis and Bax is significantly upregulated in ATDC5 cell under IL-1*β* treatment.

### 3.3. Bax Is the Direct Target of miR-29a

**Figure 3:**
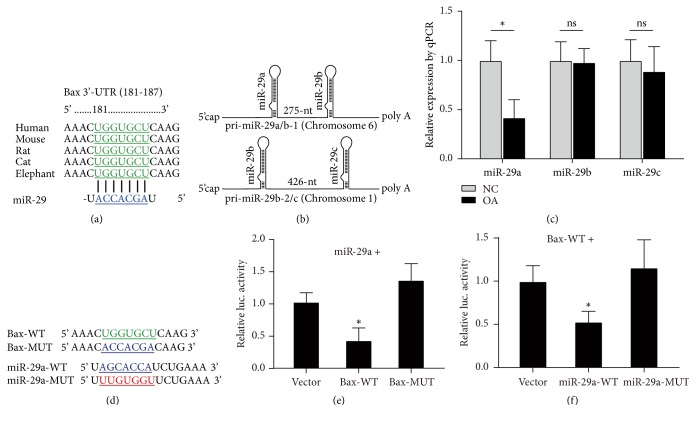
*Bax is the direct target of miR-29a*. (a) Predicted miR-29a seed matches the sequence of Bax 3'UTR. The complementary sequences are shown in green and blue as indicated. (b) Schematic representation of the genomic organization of the human miR-29. (c) Chondrocyte cell cultures of cartilage tissues from control or OA patients were subjected to quantitative PCR (qPCR) and the relative expression levels of miR-29a, miR-29b, and miR-29c were shown. *∗* donates* p* < 0.05 and ns donates no significant differences, both compared to NC group. (d) Wild type (Bax-WT) and seed mutated (Bax-MUT) 3'UTR s of Bax and mature wild type (miR-29a-WT) and seed mutated (miR-29a MUT) were shown. (e) Cultured ATDC5 cell was cotransfected with dual luciferase reporter with wild type miR-29a together with Bax-WT or Bax-MUT. The relative activity was represented. *∗* donates* p* < 0.05, compared with vector control. (f) Dual luciferase activity assays by cotransfection with wild type Bax 3'UTR together with miR-29a-WT or miR-29a-MUT expression plasmids. *∗* donates* p* < 0.05, compared with vector control.

Bax is important for IL-1*β* induced chondrocyte apoptosis; however, the regulation mechanisms of Bax in OA remain to be determined. Bioinformatical analysis tool (Targetscan) shows that Bax is a potential target of miR-29. The miR-29 binding site in the Bax 3'UTR is conserved among many vertebrates, such as human, mouse, rat, cat, and elephant (Figure 3(a)). The miR-29 family in human consists of miR-29a, miR-29b, and miR-29c, differing in only two or three bases. The genomic organization of human miR-29 was represented in Figure 3(b). We determined the expression level of miR-29 in cultured cells from control or OA patients. The quantitative PCR result showed that, in OA patients, only the level of miR-29a was significantly decreased, while miR-29b and miR-29c remained unchanged (Figure 3(c)). To determine whether Bax is the direct target of miR-29a, luciferase assay was performed. We constructed luciferase reporter containing wild type (Bax-WT) or mutated (Bax-MUT) 3'UTR of Bax and expression vectors of wild type (miR-29a-WT) or seed mutated (miR-29a-MUT) miR-29a (Figure 3(d)). We cotransfected cells with luciferase reporter of Bax-WT or Bax-MUT with miR-29a. The result showed that miR-29a reduced luciferase activity of Bax-WT, whereas miR-29a failed to affect the luciferase activity of Bax-MUT (Figure 3(e)). Cotransfection of wild type 3'UTR of Bax and miRNA (miR-29a-WT or miR-29a-MUT) expression vector showed that miR-29a-WT transfection significantly reduced the level of luciferase activity, but not miR-29a-MUT with seed mutation (Figure 3(f)). Taken together, these data demonstrate that Bax is the direct target of miR-29a.

### 3.4. miR-29a/Bax Axis Contributes to IL-1*β* Induced Chondrocyte-Like ATDC5 Apoptosis

**Figure 4:**
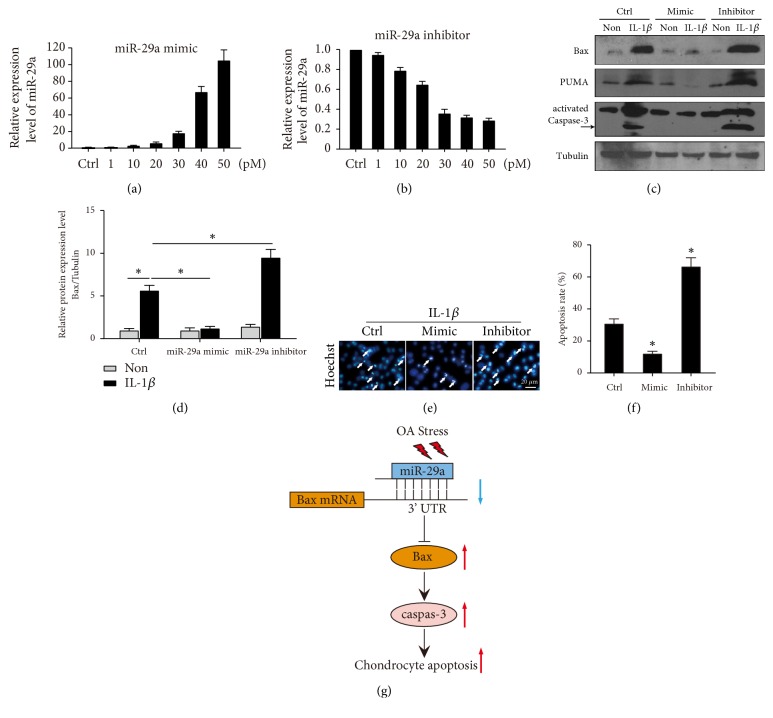
*miR-29a/Bax axis contribute to IL-1β induced chondrocyte-like ATDC5 cell apoptosis*. Cells were transfected with dose-dependent of miR-29a mimic (a) or inhibitor (b). The response levels of miR-29a were determined. (c) Representative immunoblots show Bax, PUMA, and cleaved Caspase-3 levels in IL-1*β* induced chondrocyte-like ATDC5 cell with or without miR-29a mimic or inhibitor. (d) The relative expression levels of Bax were shown. *∗* donates* p* < 0.05, compared with expression level under IL-1*β* treatment in control group. (e) Cell apoptosis was determined by Hoechst staining in cells transfected with or without miR-29a mimic/inhibitor under IL-1*β* treatment. The apoptotic rates were shown in (f). (g) Graphic abstract showing the summary of the hypothesis.

Our preliminary experiments transfected cells with miR-29a plasmids (vector or seed mutant). Since transfection with plasmid was variable than nucleotide, thus, we synthesized miR-29a mimic and inhibitor. We determined the efficiency and nontoxic concentration for transfection in ATDC5 cells and found that 30 pmol mimic or inhibitor was appropriate for transfection without overt toxicity (Figures 4(a) and 4(b)); and 30 pmol was used for subsequent experiment.

After demonstration of direct regulation of miR-29a to Bax, we tested the role of miR-29a/Bax in IL-1*β* induced chondrocyte-like ATDC5 apoptosis. Cultured ATDC5 cells were transfected with miR-29a mimic or inhibitor, or control. Then, cells were treated with or without (Non) 10 ng/ml IL-1*β* for 48 h to induce chondrocyte-like ATDC5 cell apoptosis. Bax protein level was significantly increased under IL-1*β* in control group and miR-29a mimic decreased and miR-29a inhibitor increased Bax protein levels markedly, comparing to the corresponding control (Figures 4(c) and 4(d)). Furthermore, the protein levels of PUMA and cleaved caspase-3 showed the same trend as Bax (Figure 4(c)). To test if altering miR-29a levels affect IL-1*β* induced ATDC5 apoptosis, miR-29a mimics or inhibitors were transfected into ATDC5 cells. Apoptotic assay was performed, and the results revealed that miR-29a mimics decreased while miR-29a increased significantly the apoptosis rate (Figures 4(e) and 4(f)). Collectively, these data indicate that miR-29a/Bax axis plays an important role in IL-1*β* induced chondrocyte-like ATDC5 apoptosis.

## 4. Discussion

In this study, we demonstrated that expression level of Bax was increased in cartilage tissues of OA patients and in cultured human OA chondrocyte. Furthermore, administration of IL-1*β* significantly induced Bax and PUMA upregulation, activation of Caspase-3, and finally apoptosis of chondrocyte-like ATDC5 cells. Expression level of miR-29a was markedly downregulated with in OA patients. Bax was identified as the direct effect target of miR-29a during IL-1*β* induced ATDC5 apoptosis. Modulation of miR-29a controlled the expression levels of Bax and PUMA, activation of Caspase-3, and cell apoptosis. The graphic schematic diagram shows the summary of our hypothesis (Figure 4(g)). Taken together, we specified the important role of miR-29a/Bax axis in the regulation chondrocyte apoptosis in OA.

OA is the most common disease of articular cartilage, where loss and abnormal remodeling of the matrix occurred [33]. Chondrocyte is the only cell type in the articular cartilage and chondrocyte death has been considered as a possible mechanism of OA pathology [34]. Studies showed that apoptosis occurred more frequently in OA cartilage than in normal cartilage [5, 35]. And chondrocyte apoptotic rate was positively correlated to cartilage degradation/severity of OA. However, revealed by TUNEL assay, the apoptotic rate varies from 6% [36] and 19% [5, 37] to 88% [38]. Furthermore, a recent study showed that chondrocyte death reduces catabolic cartilage damage in murine posttraumatic OA [39]. Thus, the relationship between chondrocyte loss and cartilage degeneration needs to be further explored.

Bax is one of the apoptotic executors belonging to the BH3-only subgroup of Bcl-2 family proteins [40, 41]. Upon apoptotic stimuli, Bax can be activated and oligomerized into the mitochondrial outer membrane and induce the release of Cytochrome c and activate caspase proteins [42]. Bax regulation has been implicated in many pathological processes such as neuronal degenerative disease and cancer [43]. However, the role of Bax in chondrocyte apoptosis in human OA remains largely unknown. Compared to OA lesional area in cartilage tissues from the same patient, the level of Bax remained unchanged in nonlesional area [35]. In 2015, Karaliotas GI and et al. reported that Bax mRNA level shows an increasing trend in OA patients without statistically significance; however, the level of Bax was markedly increased in stage III OA patients [44]. Thus, the role of Bax expression in OA is revealing. In the current study, we collected cartilage tissues from OA patients and analyzed the protein expression levels of Bax. By immunohistochemical and western blot, Bax expression level was found significantly upregulated in OA samples. Although we did not distinguish the OA stage of our patients, the age distribution was found close to that in the report, indicating the potential consistence [44].

On the other side, the ratio of Bcl-2/Bax is critical in regulating cell apoptosis. Bcl-2 is an antiapoptotic Bcl-2 family protein, which is consistently downregulated in the apoptosis process [45], leading to the decreased Bcl-2/Bax during cell apoptosis. The imbalance of Bcl-2/Bax also occurred in OA animal model and in cartilage tissues from OA patients [44, 46, 47]. We found that the levels of Bcl-2 proteins showed no changes between normal cartilage and OA samples (Figure 1) and Bcl-2 expression was downregulated in IL-1*β* induced ATDC5 apoptosis (Figure 2). Thus, the dysregulated Bcl-2/Bax ratio was found to contribute to chondrocyte apoptosis in the current study. Furthermore, Bax/Bcl-2 ratio imbalance has been reported to mediate miR-29a induced fibroblast apoptosis [48] and Bax has been determined as a direct target of miR-29b in myocardial apoptosis [49]. Moreover, miR-29b targets a variety of BH3-only genes, including Bim, Bmf, Hrk, Puma, and N-Bak, to regulation neuronal maturation and apoptosis [50]. However, the regulation mechanism of Bax in OA development remains unclear. Bioinformatic analysis revealed the potential regulation of miR-29 toward Bax, and by luciferase reporter assay we confirmed that Bax was the direct target of miR-29a and the miR-29a/Bax axis contributed to the regulation of IL-1*β* induced ATDC5 apoptosis.

Human miR-29 family consists of three members, miR-29a, miR-29b, and miR-29c [51]. Due to the homogenous sequences, they share overlapping target. However, differed roles have been reported [52]. In regulation OA, all three miR-29s have been reported to increase in human hip OA samples, whereas downregulated miR-29s have been found in cultured human OA chondrocyte [53]. Additionally, miR-29c is increased in the plasma of patients with primary OA [54]. Moreover, miR-29a is downregulated in human hip and knee OA articular cartilage and shows a negative correlation of miR-29a with body mass index (BMI) while IL-1*β* positively correlates with BMI [29], indicating the inhibition effect of IL-1*β* on miR-29a expression in OA. Further, miR-29a is downregulated and miR-29a deficiency exacerbates synovitis pathogenesis and fibrosis in the end-stage of OA knees [55]. In the current study, miR-29a was downregulated in OA cartilage samples, resulting in the upregulation of miR-29a targeted Bax and ultimately chondrocyte apoptosis. Thus, the role of miR-29 in OA is revealing and the results differ because of individual differences of collected samples. Large scale of OA cartilage tissues may be needed to further identify the role of miR-29a in the development of OA.

In summary, the present study determines that miR-29a/Bax axis plays an important role in chondrocyte apoptosis in OA. Upon OA stimuli, the levels of miR-29a are downregulated and Bax expression is increased, leading to the activation of Caspase-3 protein and finally chondrocyte apoptosis. These findings provide new evidence for the understanding of OA development and new strategies for OA caring in the future.

## Data Availability

The data used to support the findings of this study are available from the corresponding author upon request.

## Abbreviations

DAB: Diaminobenzidine tetrahydrochloride
DMEM: Dulbecco's Modified Eagle Medium
FBS: Fetal bovine serum
IL-1*β*: Interleukin-1beta
OA: Osteoarthritis
PAGE: SDS-polyacrylamide gel electrophoresis.
